# Supplementary material for: An evolutionary timeline of the oxytocin signaling pathway
Source: Commun Biol. 2024 Apr 17;7:471. doi: 10.1038/s42003-024-06094-9 (PMC11024182; doi:10.1038/s42003-024-06094-9)
Supplement: Supplementary file 2 — Supplementary Information [file 42003_2024_6094_MOESM2_ESM.pdf]

Supplementary figure 1

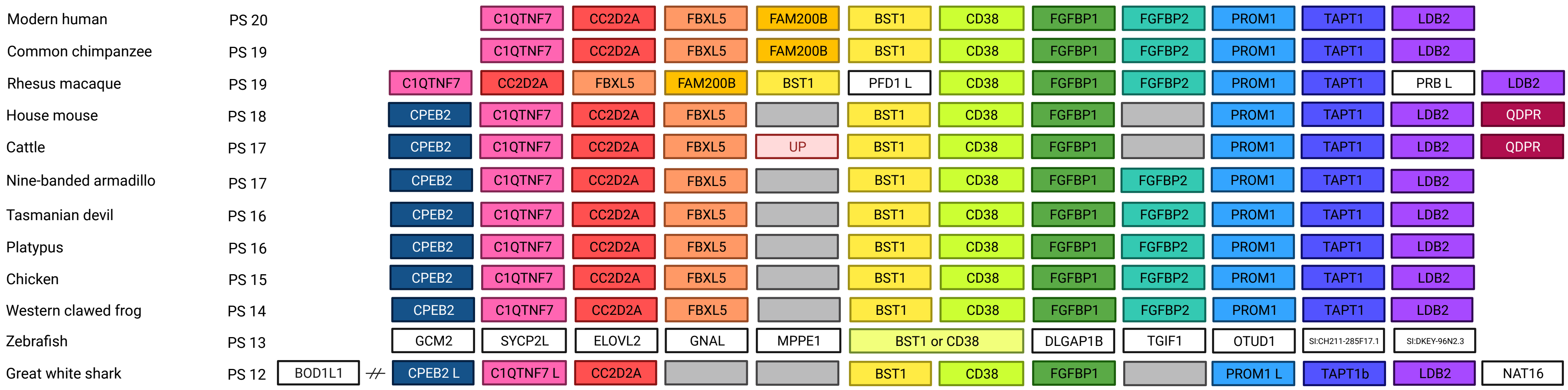

Microsynteny for *CD38* across PS 20 to 12. The ten genes surrounding *CD38* in the modern human build a microsyntenic block that is well conserved in a tetrapode species (PS 14). The synteny is slightly more scattered in more ancient species like the great white shark, and ostensibly not present in the zebrafish. This suggests that the syntenic block that can be observed in humans first set in the tetrapode ancestor but might have roots dating back to the gnathostome ancestor. The species' common names are given in the outer left column, followed by the corresponding PS and the genes and microsyntenic block. Each rectangle represents one gene with the abbreviated gene name in the center. Grey, empty rectangles indicate a potential missing gene in that locus. PS = Phylostratum; UP = Uncharacterized protein.

Supplementary figure 2

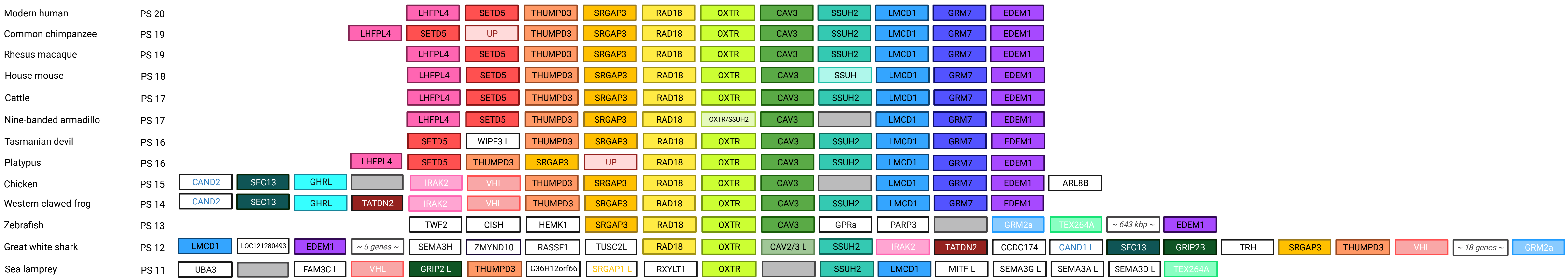

Microsynteny for *OXTR* across PS 20 to 12. The ten genes surrounding *OXTR* in the modern human build a microsyntenic block that is conserved across mammalian species (PS20-16). The synteny is slightly more scattered in amniotes and tetrapodes. The synteny is visibly scattered in the zebrafish and more ancient species, and the syntenic block seems to be almost absent in the first vertebrates (e.g., sea lamprey), although traces of it are discernible. This suggests that the syntenic block that can be observed in humans first set in the mammalian ancestor, and started forming in the first vertebrates. The species' common names are given in the outer left column, followed by the corresponding PS and the genes and microsyntenic block. Each rectangle represents one gene with the abbreviated gene name in the center. Grey, empty rectangles indicate a potential missing gene in that locus. PS = Phylostratum; UP = Uncharacterized protein.

Manual for positive selection analysis in Sartorius et al., 2024 (https://doi.org/10.31219/osf.io/42b8g)

This manual is written for the somewhat **naïve user** that does **not** have a background in genetics, biology, informatics, evolution, etc. but rather in neuro-/cognitive-/biological- psychology, medicine, etc. and a basic education in statistics and coding.

Abbreviations

OS = operating system
dir = directory
CDS = coding sequence
cd = navigate to, go to
AAS = amino acid sequence, protein sequence
MSA = multiple sequence alignment

Preparations – system and program requirements

This manual is written for a Mac OS (Ventura 13.2.1 or newer) with access to a virtual Windows environment. If not otherwise specified, all the installations are done for Mac. The manual assumes that the user has pre-installed R and R Studio, the newest versions available.

NB: If the user uses this manual as part of re-creating all the analyses from the paper (e.g., BLASTp, microsynteny, cerebral gene expression), it is strongly recommended – unless stated otherwise – that the user operates from within the directory "OTpathway_evolution_main" that has been previously created as described in the "README_OTevo", and downloads/generates all files, folders, programs, etc. in that directory.

NB: For the user aiming only to perform the positive selection analysis, it most of the times does not matter too much where programs/executables are downloaded to/located as long as you *know* where they are located on your machine (i.e., you know the full path to the file). However, it often does make things easier to have everything in one place or dir.

1. Install Miniconda3

- Download: https://repo.anaconda.com/miniconda/Miniconda3-latest-MacOSX-arm64.sh
- Execute `sh <PATH-TO-FILE/Miniconda3-latest-MacOSX-arm64.sh>` in terminal

DONE

2. Install homebrew

- Execute `/bin/bash -c "$(curl -fsSL https://raw.githubusercontent.com/Homebrew/install/HEAD/install.sh)"` In the terminal

DONE

3. Install MUSCLE (on windows computer)

- Go to https://github.com/rcedgar/muscle/releases/tag/5.1.0 and download the "muscle5.1.win64.exe" executable file
- Rename muscle5.1.mac…. to "muscle"

DONE

4. Install PAL2NAL

- In the terminal, execute `conda install -c bioconda pal2nal`

DONE

5. Install TriFusion

- Go to https://odiogosilva.github.io/TriFusion/ and download the zip file for MacOS
- Also download the user manual – it can be very helpful
- Unzip the file
- Open terminal and cd to the dir where the app is located
- Execute the following command in terminal, this gives executing permission to TriFusion `chmod +x TriFusion.app/Contents/MacOS/TriFusion`

DONE

6. Install Xcode Developer Tools

- Go to the App Store, search for the app and install the newest version

DONE

7. Install Command Line Tools for Mac

- Go to https://developer.apple.com/download/all/ and choose "Command Line Tools for Xcode 14.3.1" or whichever version of Xcode you have installed on your Mac
- Download the .dmg file and open it. Follow the installation instructions

DONE

8. Install CMake (necessary for HyPhy)

- You can install cmake either via homebrew by executing `brew install cmake` in your terminal OR from source (both options worked for me on my Mac)
- For the source installation download the newest source distributions at www.cmake.org/download/ (e.g., "cmake-3.27.1.tar.gz") and unzip them
- Cd into the unzipped cmake compilation folder
- In the terminal, execute `./bootstrap` (this may take a while)
- Then execute `make` (this may take a while, too)
    - Online you might find that it says to run "make install" **but that is optional**
- Check whether it worked by running `cmake`, `cmake --version` and `cmake --help`

DONE

9. Install HyPhy

- Create a folder on your computer called "HYPHY" (all capital letters)
- In the terminal, cd into that folder
- Execute `git clone https://github.com/veg/hyphy.git`
- Cd into the folder "hyphy" you just created/cloned and execute `cmake .`

  NB: It might say things like "Could NOT find OpenMP_C", Could NOT find OpenMP", "Could NOT find MPI_C", etc. while the compiler runs. That is **normal**, don't worry. The important thing is that it says "Configuring done", "Generating done" and "Build files have been written to: some/path/on/you/computer/HYPHY/hyphy" at the end. **Remember** that last path where the build files have been written to, you will need the path later during the installation.
- Stay in the dir where you are and execute `make` (just `make`, **NOT** `make install`)

- It will build CXX objects and show you the progress in percentages. At the end it should say “[100%] Linking CXX executable hyphy” “mklink HYPHYMP -> hyphy” “[100%] Built target hyphy”

- Cd into the HYPHY dir with `cd ..`
- Set library path to the written files by execute `HYPHY/hyphy LIBPATH= some/path/on/you/computer/from/earlier/that/you/were/supposed/to/remember/HYPHY/hyphy/res`
- Check whether it worked by executing `./hyphy –help` and `./hyphy --version`

DONE

Test for positive selection in PAML

(for Mac with access to virtual Windows environment)

1. Download sequences

- Manually download the orthologous AAS of each gene for each species. To assure selection of credible orthologous sequences, open the supplementary data 2 (see https://osf.io/rxphw). Go through the relevant 39 sheets (each gene has its own sheet with the vertebrate microsynteny, but you only want to focus on the 39 genes of interest here): *ADCY1*, ADCY2, *ADCY7*, *ADCY8*, *ADCY9*, *CACNA2D1*, *CACNA2D3*, *CACNA2D4*, *CACNG1*, *CACNG3*, *CACNG4*, *CACNG5*, *CD38*, *EEF2K*, *EGFR*, *ELK1*, *FOS*, *GUCY1A2*, *JUN*, *KCNJ2*, *KCNJ3*, *KCNJ4*, *KCNJ5*, *KCNJ12*, *MEF2C*, *MYLK3*, *NFATC1*, *NFATC2*, *NFATC3*, *OXT*, *OXTR*, *PIK3R5*, *PLA2G4A*, *PLA2G4F*, *PLCB1*, *PPP1R12A*, *PRKAB1*, *RAF1*, *RYR3*. In each sheet there is a “START” column where the accession numbers for the target gene in each species are provided as a comment/note. Search for those accession numbers in NCBI (https://ncbi.nlm.nih.gov/) in the “gene” database and you will be guided to an overview page for the gene in the species. Scroll down to the “NCBI Reference Sequences (RefSeq)” section, subsection “mRNA and Protein(s)”, where you will find the correct AAS listed together with the corresponding CDS (each AAS is tied to a specific CDS/nucleotide sequence, for instance the AAS for *OXT* in humans “NP_000906.1” is tied to the CDS “NM_000915.4”).

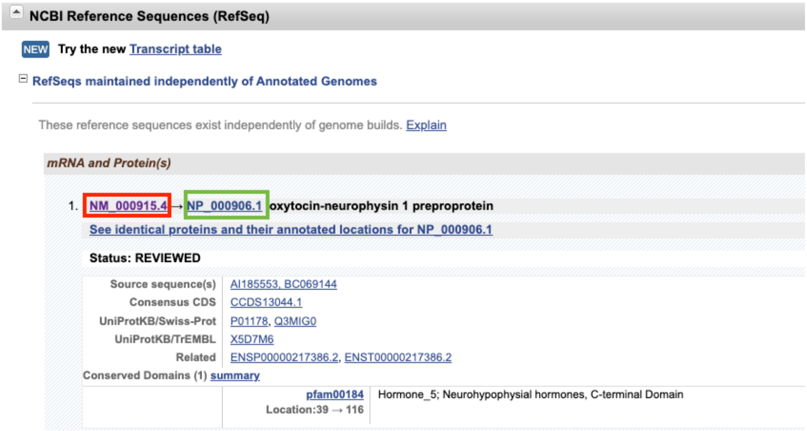

- Click on the AAS, which will redirect you to another page where you click ”FASTA”, then “Send to: ∨” → Select “File” → Select “Format – FASTA” → Click “Create File”. Like this you download each AAS in FASTA format.
- Repeat the same for the corresponding CDS by clicking on the CDS, which will redirect you to another page where you click ”FASTA”, then “Send to: ∨” → Select “Coding Sequences” → Select “Format – FASTA Nucleotide” → Click “Create File”. Like this you download each CDS in FASTA format.

- For each gene, aggregate all the AAS from each species into a large file, named "gene_proseq.fasta", e.g., for *OXT* gather the 11 orthologous AAS from the 11 species into one file called "oxt_proseq.fasta".
- Repeat the same for the CDS naming each aggregated file "gene_nucseq.fasta", e.g., oxt_nucseq.fasta, adcy1_nucseq.fasta, etc.
- I did this aggregation/concatenation manually, that means I copy-pasted each sequence into one gene file one by one but you can of course use a script or a program for this. A program could be TriFusion, or command line scripts for this are provided by Álvarez-Carretero et al., 2023 (https://doi.org/10.1093/molbev/msad041, https://github.com/abacus-gene/paml-tutorial/tree/main/positive-selection/00_data)

- For comparison, the files I downloaded (some already renamed, cf. step 3) are available on OSF (https://osf.io/rxphw/, directories `../Code and data/scripts_files_positive_selection_analysis/step01_files/00nucseqs/`"
 and
 `../Code and data/scripts_files_positive_selection_analysis/step01_files/00nproseqs/`)

2. Align **protein** sequences (create protein MSA)
 For each gene, align the AAS from a given aggregated FASTA file. Right now, the AAS are just randomly gathered in an aggregated FASTA file without structure or meaning. We need **`MUSCLE`** for that, see above for how to install it on *Windows* (I unfortunately did not manage to install it on Mac. If you succeed you can of course run it on a Mac OS.)
 - On your virtual Windows environment create a "mother" working directory (mwd) and in that mwd create the following folders: *00proseqs* (folder for your 'raw' protein sequences) and *01proseqs_align* (folder for the to-be-aligned protein sequences)
 - Copy the aggregated AAS FASTA files to the 00proseqs folder in the virtual Windows environment
 - Navigate to the head dir where the FASTA files are stored, adjust paths as needed
 ```
 cd \\path\to\the\mwd\
 ```
 and then execute
 ```
 \\path\to\muscle.exe -align .\00proseqs\oxt_proseq.fasta -output
 .\01proseqs_align\oxt_proseq_outv2.fasta
 ```
 in Windows PowerShell.

 → **Repeat for the remaining genes**

 NB: Important – Remember to copy the protein MSA files from the virtual Windows environment to your Mac when you are done

3. Translate into codon-based alignments
 a. Manually change the identifier (the lines starting with ">….." before the beginning of the actual sequences) of each AAS in the 39 protein MSA files to the following pattern: ">CDSaccessionnumber_AASaccessionnumber_gene_speciesabbreviation", e.g., ">NM_000915.4_NP_000906.1_oxt_hsapiens". Change the identifier of each CDS in the 39 aggregated CDS FASTA files to the same pattern

 NB: Make sure that an AAS in a protein MSA file shares a **unique** identifier/name with the corresponding CDS in the CDS file, i.e., it should be identical in the protein MSA files and the CDS files. For instance in "oxt_proseq.fasta" and "oxt_nucseq.fasta", both the *OXT* AAS and the *OXT*

CDS, respectively, for the modern human should be called ">NM_000915.4_NP_000906.1_oxt_hsapiens", see example:

MSA for *OXT*

```
>XM_004464296.2_XP_004464353.1_oxt_dnovemcinctus
MT-------SSQDG-EPQV--EKLAGASWIYSEPRNRSQALLI-SNMQLKCLPCGPGGRGRCFGPGICCAAELGCFVGTA
EALRCQDESRLPSPCQSGPQPCGS-GGRCAAPGICCSPDGCRTHPACDP-DAA---------------------------
FSQL
>NM_000915.4_NP_000906.1_oxt_hsapiens
MA-------GPSLA-CCLLGLLALTSACYIQNCPLGGKRAAPD-LDV-RKCLPCGPGGKGRCFGPNICCAEELGCFVGTA
EALRCQEENYLPSPCQSGQKACGS-GGRCAVLGLCCSPDGCHADPACDA-EAT---------------------------
FSQR
>NM_011025.4_NP_035155.1_oxt_mmusculus
MA-------CPSLA-CCLLGLLALTSACYIQNCPLGGKRAVLD-LDM-RKCLPCGPGGKGRCFGPSICCADELGCFVGTA
EALRCQEENYLPSPCQSGQKPCGS-GGRCAATGICCSPDGCRTDPACDP-ESA---------------------------
FSER
>NM_176855.1_NP_789825.1_oxt_btaurus
MA-------GSSLA-CCLLGLLALTSACYIQNCPLGGKRAVLD-LDV-RTCLPCGPGGKGRCFGPSICCGDELGCFVGTA
EALRCQEENYLPSPCQSGQKPCGS-GGRCAAAGICCSPDGCHEDPACDP-EAA---------------------------
FSQH
```

CDS for *OXT*

```
>NM_176855.1_NP_789825.1_oxt_btaurus
ATGGCAGGTTCCAGCCTCGCCTGCTGCCTGCTCGGCCTCCTGGCGTTGACCTCCGCCTGCTACATTCAGA
ACTGCCCCCTGGGCGGCAAACGCGCGGTGCTGGACCTCGACGTGCGCACGTGTCTCCCCTGCGGCCCCGG
GGGCAAAGGCCGCTGCTTCGGGCCCAGCATCTGCTGCGGGGACGAGCTGGGCTGCTTCGTGGGCACGGCC
GAGGCGCTGCGCTGCCAAGAGGAGAACTACCTGCCGTCGCCCTGCCAGTCCGGCCAGAAGCCCTGCGGGA
GCGGGGGCCGCTGCGCCGCCGCCGGCATCTGCTGCAGCCCGGACGGCTGCCACGAGGACCCCGCCTGCGA
CCCTGAGGCCGCCTTCTCCCAGCACTGA

>XM_004464296.2_XP_004464353.1_oxt_dnovemcinctus
ATGACCTCCTCTCAAGATGGAGAGCCCCAAGTAGAGAAGCTCGCGGGGGCCTCCTGGATCTACTCTGAGC
CTAGAAACAGGTCACAGGCATTGCTTATTTCCAATATGCAATTAAAGTGCCTGCCCTGCGGCCCCGGGGG
CAGAGGGCGCTGCTTCGGGCCCGGCATCTGCTGCGCCGCCGAGCTGGGCTGCTTCGTGGGCACGGCCGAG
GCGCTGCGCTGCCAGGACGAGAGCCGCCTGCCGTCGCCCTGCCAGTCGGGCCCGCAGCCGTGCGGGAGCG
GGGGCCGCTGCGCCGCGCCCGGCATCTGCTGCAGCCCGGACGGCTGCCGCACGCACCCTGCCTGCGACCC
CGACGCCGCCTTTTCCCAGCTCTGA

>NM_000915.4_NP_000906.1_oxt_hsapiens
ATGGCCGGCCCCAGCCTCGCTTGCTGTCTGCTCGGCCTCCTGGCGCTGACCTCCGCCTGCTACATCCAGA
ACTGCCCCCTGGGAGGCAAGAGGGCCGCGCCGGACCTCGACGTGCGCAAGTGCCTCCCCTGCGGCCCCGG
GGGCAAAGGCCGCTGCTTCGGGCCCAATATCTGCTGCGCGGAAGAGCTGGGCTGCTTCGTGGGCACCGCC
GAAGCGCTGCGCTGCCAGGAGGAGAACTACCTGCCGTCGCCCTGCCAGTCCGGCCAGAAGGCGTGCGGGA
GCGGGGGCCGCTGCGCGGTCTTGGGCCTCTGCTGCAGCCCGGACGGCTGCCACGCCGACCCTGCCTGCGA
CGCGGAAGCCACCTTCTCCCAGCGCTGA

>XM_002936359.2_XP_002936405.1_oxt_xtropicalis
ATGACCTACAGCACCCTGGCCGCGTGTCTCTTCTGCCTTCTGGCTCTCACTTCAGCTTGCTACATCCAAA
ACTGCCCCATTGGAGGAAAGAGATCGGTGATGGACATCATGGACATCAGGAAGTGTATCCCTTGTGGGCC
```

→ This step improves organization of the sequences. **Further, it is necessary to name the sequences in exactly that pattern otherwise a later analysis step which uses Regex expressions will not work.** Lastly, it prevents you from losing track of all the sequences and helps you to easily identify which AAS and which CDS belong together.

b. Re-order the sequences in the CDS files so that they match the order of the sequences in the corresponding protein MSA files, e.g., the order of the sequences in oxt_nucseq.fasta should be re-organized so that they match the order of the aligned AAS in oxt_proseq_outv2.fasta

NB: This step is ***strictly necessary*** and ***essential*** for PAL2NAL to work!

Example for the first five sequences in *OXT*

CDS

```
>XM_004464296.2_XP_004464353.1_oxt_dnovemcintus 1
ATGACCTCCTCTCAAGATGGAGAGCCCCAAGTAGAGAAGCTCGCGGGGGCCTCCTGGATCTACTCTGAGC
CTAGAAACAGGTCACAGGCATTGCTTATTTCCAATATGCAATTAAAGTGCCTGCCCTGCGGCCCCGGGGG
CAGAGGGCGCTGCTTCGGGCCCGGCATCTGCTGCGCCGCCGAGCTGGGCTGCTTCGTGGGCACGGCCGAG
GCGCTGCGCTGCCAGGACGAGAGCCGCCTGCCGTCGCCCTGCCAGTCGGGCCCGCAGCCGTGCGGGAGCG
GGGGCCGCTGCGCCGCGCCCGGCATCTGCTGCAGCCCGGACGGCTGCCGCACGCACCCTGCCTGCGACCC
CGACGCCGCCTTTTCCCAGCTCTGA

>NM_000915.4_NP_000906.1_oxt_hsapiens 2
ATGGCCGGCCCCAGCCTCGCTTGCTGTCTGCTCGGCCTCCTGGCGCTGACCTCCGCCTGCTACATCCAGA
ACTGCCCCCTGGGAGGCAAGAGGGCCGCGCCGGACCTCGACGTGCGCAAGTGCCTCCCCTGCGGCCCCGG
GGGCAAAGGCCGCTGCTTCGGGCCCAATATCTGCTGCGCGGAAGAGCTGGGCTGCTTCGTGGGCACCGCC
GAAGCGCTGCGCTGCCAGGAGGAGAACTACCTGCCGTCGCCCTGCCAGTCCGGCCAGAAGGCGTGCGGGA
GCGGGGGCCGCTGCGCGGTCTTGGGCCTCTGCTGCAGCCCGGACGGCTGCCACGCCGACCCTGCCTGCGA
CGCGGAAGCCACCTTCTCCCAGCGCTGA

>NM_011025.4_NP_035155.1_oxt_mmusculus 3
ATGGCCTGCCCCAGTCTCGCTTGCTGCCTGCTTGGCTTACTGGCTCTGACCTCGGCCTGCTACATCCAGA
ACTGCCCCCTGGGCGGCAAGAGGGCTGTGCTGGACCTGGATATGCGCAAGTGTCTCCCCTGCGGCCCGGG
CGGCAAAGGACGCTGCTTCGGACCAAGCATCTGCTGCGCGGACGAGCTGGGCTGCTTCGTGGGCACCGCC
GAGGCGCTGCGCTGCCAGGAGGAGAACTACCTGCCTTCGCCCTGCCAGTCTGGCCAGAAGCCCTGCGGGA
GCGGAGGCCGCTGCGCCGCCACAGGCATCTGCTGCAGCCCGGATGGCTGCCGCACAGACCCCGCCTGCGA
CCCTGAGTCTGCCTTCTCGGAGCGCTGA

>NM_176855.1_NP_789825.1_oxt_btaurus 4
ATGGCAGGTTCCAGCCTCGCCTGCTGCCTGCTCGGCCTCCTGGCGTTGACCTCCGCCTGCTACATTCAGA
ACTGCCCCCTGGGCGGCAAACGCGCGGTGCTGGACCTCGACGTGCGCACGTGTCTCCCCTGCGGCCCCGG
GGGCAAAGGCCGCTGCTTCGGGCCCAGCATCTGCTGCGGGGACGAGCTGGGCTGCTTCGTGGGCACGGCC
GAGGCGCTGCGCTGCCAAGAGGAGAACTACCTGCCGTCGCCCTGCCAGTCCGGCCAGAAGCCCTGCGGGA
GCGGGGGCCGCTGCGCCGCCGCCGGCATCTGCTGCAGCCCGGACGGCTGCCACGAGGACCCCGCCTGCGA
CCCTGAGGCCGCCTTCTCCCAGCACTGA

>XM_040671655.2_XP_040527589.1_oxt_ggallus 5
ATGTTCTACAAGGCGCTCACCGTCTGCCTGCTGGGGCTTCTGGCTCTCTCCTCAGCTTGTTATATCCAGA
ACTGCCCCATCGGGGGTAAGCGTGCCGTGCCGGACATGGACATCAGGAAGTGCCTGCCCTGCGGCCCCAG
GAACAAGGGCCACTGCTTTGGCCCCAACATCTGCTGCGGGGAGGAGCTGGGCTGCTACCTGGGCACCTCT
GAAACCCTGCGGTGCCAGGAGGAGAACTTCCTGCCAACGCCCTGTGAGTCTGGGAGGAAAGCCTGCGGCG
AGGATGGGGCGAGCTGTGCAGCACCAGGAATCTGCTGCAGCAGTGAGGGCTGTGTGCTTGACTCATCTTG
CAACCAAGAAATGCTGTTTGCATAG
```

MSA

```
>XM_004464296.2_XP_004464353.1_oxt_dnovemcintus 1
MT-------SSQDG-EPQV--EKLAGASWIYSEPRNRSQALLI-SNMQLKCLPCGPGGRGRCFGPGICCAAELGCFVGTA
EALRCQDESRLPSPCQSGPQPCGS-GGRCAAPGICCSPDGCRTHPACDP-DAA---------------------------
FSQL
>NM_000915.4_NP_000906.1_oxt_hsapiens 2
MA-------GPSLA-CCLLGLLALTSACYIQNCPLGGKRAAPD-LDV-RKCLPCGPGGKGRCFGPNICCAEELGCFVGTA
EALRCQEENYLPSPCQSGQKACGS-GGRCAVLGLCCSPDGCHADPACDA-EAT---------------------------
FSQR
>NM_011025.4_NP_035155.1_oxt_mmusculus 3
MA-------CPSLA-CCLLGLLALTSACYIQNCPLGGKRAVLD-LDM-RKCLPCGPGGKGRCFGPSICCADELGCFVGTA
EALRCQEENYLPSPCQSGQKPCGS-GGRCAATGICCSPDGCRTDPACDP-ESA---------------------------
FSER
>NM_176855.1_NP_789825.1_oxt_btaurus 4
MA-------GSSLA-CCLLGLLALTSACYIQNCPLGGKRAVLD-LDV-RTCLPCGPGGKGRCFGPSICCGDELGCFVGTA
EALRCQEENYLPSPCQSGQKPCGS-GGRCAAAGICCSPDGCHEDPACDP-EAA---------------------------
FSQH
>XM_040671655.2_XP_040527589.1_oxt_ggallus 5
MF-------YKALT-VCLLGLLALSSACYIQNCPIGGKRAVPD-MDI-RKCLPCGPRNKGHCFGPNICCGEELGCYLGTS
ETLRCQEENFLPTPCESGRKACGEDGASCAAPGICCSSEGCVLDSSCNQ-EML---------------------------
FA--
[illegible]
```

→ **Note that the CDS are in the same order as the aligned AAS file**

c. Execute the following code in the terminal, **repeat for all genes** (below is an example for the gene *ADCY1*). Adjust the paths as needed. The code can be executed from anywhere, no need to cd into a specific dir.

For FASTA output format:

```
pal2nal.pl
/path/to/folder/with/aligned/AAS/on/your/Mac/adcy1_proseq_outv2.fasta
/path/to/folder/with/aggregated/unaligned/CDS/files/on/your/Mac/adcy1_nucseq.fasta -output fasta >
/path/to/output/dir/where/to/store/results/adcy1_codalign.fa
```

→ **This way you get codon-based aligned <u>nucleotide</u> sequences for each gene which we need for HyPhy/aBSREL later**

NB: If you accidentally match a protein MSA and a CDS file that do not belong together, f.i., *adcy1_proseq_outv2.fasta* and *oxt_nucseq.fasta*, the program will result in an error. It is impossible to match the wrong files and not notice it.

NB: Remember to adjust the name of the output file with every run so you don't overwrite files you have generated before

4. <u>Missing sequences</u>
Some genes do not have orthologs in all species. For instance, most genes do not have an ortholog in *P. marinus*, some not in *D. novemcinctus*, etc. Thus, in some gene files, there are only 10 sequences, while in others there are 13. What I did is add empty dummy sequences to some gene files. **NB:** The number of hyphons "-" has to be the same length as the original sequence length in the MSA! This is crucial, **otherwise the final analysis will not run**.
For instance, in the aligned FASTA file for *OXT*, there are only 11 sequences, there was no credible ortholog in *P. marinus* or *S. harrisii*. I added the following two empty sequences to indicate a missing ortholog:

```
>XM_00000.0_XP_00000.0_oxt_sharrisii
------------------------------------------------------------
------------------------------------------------------------
------------------------------------------------------------
------------------------------------------------------------
------------------------------------------------------------
------------------------------------------------------------
------------------------------------------------------------
------------------------------------------------------------
------------
```

and

```
>XM_00000.0_XP_00000.0_oxt_pmarinus
------------------------------------------------------------
------------------------------------------------------------
------------------------------------------------------------
------------------------------------------------------------
------------------------------------------------------------
------------------------------------------------------------
------------------------------------------------------------
------------------------------------------------------------
------------
```

NB: Make a safety copy of the original files first and move them to a new folder called “fasta_processed”. Work with those copies.

- For comparison, the processed files are available on OSF (https://osf.io/rxphw/, directory `../Code and data/scripts_files_positive_selection_analysis/step04_files/`)

5. Preparing the MSA input file
   a. *Remove any information but taxon name from all sequences in all FASTA files.*
   Open the script “keep_taxnames_fastas.sh” (the script is available at https://osf.io/rxphw/). Customize the `source_directory` and `destination_directory` paths according to your local set up. The script will automatically create a dir “fasta_processed_taxnames” where the new files will be stored in. In the terminal, navigate into the dir where the script “keep_taxnames_fastas.sh” is stored with the `cd` command. Make the script executable with `chmod +x keep_taxnames_fastas.sh` and then execute it with `./keep_taxnames_fastas.sh`.
   NB: The script contains a Regex expression that will only work if you named your sequence identifiers according to the pattern described above. If you used a different pattern you will need to adjust the Regex expression accordingly.
   NB: Your files must have the file ending `.fa`. If they have `.fasta` you need to change the script accordingly in line 16.
   Each file should look like this in the end:

```
>hsapiens
ATGGCC---------------------GGCCCCAGCCTCGCT---TGCTGTCTGCTCGGC
CTCCTGGCGCTGACCTCCGCCTGCTACATCCAGAACTGCCCCCTGGGAGGCAAGAGGGCC
GCGCCGGAC---CTCGACGTG---CGCAAGTGCCTCCCCTGCGGCCCCGGGGGCAAAGGC
CGCTGCTTCGGGCCCAATATCTGCTGCGCGGAAGAGCTGGGCTGCTTCGTGGGCACCGCC
GAAGCGCTGCGCTGCCAGGAGGAGAACTACCTGCCGTCGCCCTGCCAGTCCGGCCAGAAG
GCGTGCGGGAGC---GGGGGCCGCTGCGCGGTCTTGGGCCTCTGCTGCAGCCCGGACGGC
TGCCACGCCGACCCTGCCTGCGACGCG---GAAGCCACC---------------------
------------------------------------------------------------
TTCTCCCAGCGC
>ptroglodytes
ATGGCC---------------------GGCCCCAGCCTCGCT---TGCTGTCTGCTCGGC
CTCCTGGCGCTGACCTCCGCCTGCTACATCCAGAACTGCCCCCTGGGAGGCAAGAGGGCC
GCGCCGGAC---CTCGACGTG---CGCAAGTGCCTCCCCTGCGGCCCCGGGGGCAAAGGC
CGCTGCTTCGGGCCCAATATCTGCTGCGCAGAAGAGCTGGGCTGCTTCGTGGGCACCGCC
GAAGCGCTGCGCTGCCAGGAGGAGAACTACCTGCCGTCGCCCTGCCAGTCCGGCCAGAAG
GCGTGCGGGAGC---GGGGGCCGCTGCGCGGTCTTGGGCCTCTGCTGCAGCCCGGACGGC
TGCCACGCCGACCCTGCCTGCGACATG---GAAGCTACC---------------------
------------------------------------------------------------
TTCTCCCAGCAC
>mmulatta
ATGGCC---------------------GGCCCCAGCCTCGCC---TGCTGTCTCCTCGGC
CTTCTGGCGCTGACCTCCGCCTGCTACATCCAGAACTGCCCCCTGGGAGGCAAGAGGGCC
GCGCCGGAC---CTCGACGTG---CGCAAGTGCCTCCCCTGCGGCCCCGGGGGCAAAGGC
CGTTGCTTTGGGCCCAATATCTGCTGCGCGGAAGAGCTGGGCTGCTTCGTGGGCACGGCC
GAGGCGCTGCGCTGCCAGGAGGAGAACTACCTGCCGTCGCCCTGCCAGTCGGGCCAGAAG
[illegible]
```

   b. *Convert from FASTA to PHYLIP* ***sequential*** *format*
   (You can also do interleaved format, does not matter for PAML)
      i) Open TriFusion by left-clicking the app, no terminal or anything else coding-wise needed.
      ii) Drag and drop all the 39 codon alignments simultaneously into TriFusion, select “Alignment” in the pop-up window and confirm. Double check everything went smoothly by going to the Menu with the three horizontal

bars in TriFusion and checking whether the right number of files and taxa are there by selecting the “Files” and “Taxa” buttons below the “Open file(s)” button

→ if something went wrong with the previous steps (too little or too many hyphens, wrong taxa name), you will get error messages here while uploading the alignments

iii) Go to the “Process” tab and select “Conversion”. Choose “Active files” in Data set, “Phylip” only in Output Format and your Output dir of choice. Ignore the additional options.

iv) Hit “Execute” and confirm with “Execute” again

The files should look like this in the end (examples for *OXT* and *FOS*):

OXT

```
13 492
hsapiens                                 atggcc---------------------ggccccagcctcgct---
tgctgtctgctcggcctcctggcgctgacctccgcctgctacatccagaactgcccccctgggaggcaagagggccgcgccggac---ctcgacgtg---
cgcaagtgcctcccctgcggccccgggggcaaaggccgctgcttcgggcccaatatctgctgcgcggaagagctgggctgcttcgtgggcaccgccgaagc
gctgcgctgccaggaggagaactacctgccgtcgccctgccagtccggccagaaggcgtgcgggagc---
gggggccgctgcgcggtcttgggcctctgctgcagcccggacggctgccacgccgaccctgcctgcgacgcg---
gaagccacc----------------------------------------------------------------------------------
ttctcccagcgc
ptroglodytes                             atggcc---------------------ggccccagcctcgct---
tgctgtctgctcggcctcctggcgctgacctccgcctgctacatccagaactgcccccctgggaggcaagagggccgcgccggac---ctcgacgtg---
cgcaagtgcctcccctgcggccccgggggcaaaggccgctgcttcgggcccaatatctgctgcgcagaagagctgggctgcttcgtgggcaccgccgaagc
gctgcgctgccaggaggagaactacctgccgtcgccctgccagtccggccagaaggcgtgcgggagc---
gggggccgctgcgcggtcttgggcctctgctgcagcccggacggctgccacgccgaccctgcctgcgacatg---
gaagctacc----------------------------------------------------------------------------------
ttctcccagcac
mmulatta                                 atggcc---------------------ggccccagcctcgcc---
tgctgtctcctcggccttctggcgctgacctccgcctgctacatccagaactgcccccctgggaggcaagagggccgcgccggac---ctcgacgtg---
cgcaagtgcctcccctgcggccccgggggcaaaggccgttgctttgggcccaatatctgctgcgcggaagagctgggctgcttcgtgggcacggccgaggc
gctgcgctgccaggaggagaactacctgccgtcgccctgccagtcgggccagaaggcgtgcgggagc---
ggcggccgctgcgccgtcttcggcctctgctgtagcccggacggctgccacgccgaccctgcctgcgatatg---
gaagctacc----------------------------------------------------------------------------------
ttctcccagcac
mmusculus                                atggcc---------------------tgccccagtctcgct---
tgctgcctgcttggcttactggctctgacctcggcctgctacatccagaactgcccccctgggcggcaagagggctgtgctggac---ctggatatg---
[illegible]
```

FOS

```
13 1257
hsapiens                                 atgatgttctcgggcttcaac------
gcagactacgaggcgtcatcctcccgctgcagcagcgcgtccccggccggggatagcctctcttactaccactcacccgcagactccttctccagcatggg
ctcgcctgtcaacgcgcaggacttctgcacggacctggccgtctccagtgccaacttcattcccacggtcactgccatctcgaccagtccggacctgcagt
ggctggtgcagcccgccctcgtctcctccgtggccccatcgcagaccagagcccctcaccctttcggagtccccgccccctcc---
gctggggcttactccagggctggcgttgtgaagaccatgacaggaggccgagcgcagagcattggcaggaggggcaaggtggaacagttatctccagaaga
agaagagaaaaggagaatccgaagggaaaggaataagatggctgcagccaaatgccgcaaccggaggagggagctgactgatacactccaagcggagacag
accaactagaagatgagaagtctgctttgcagaccgagattgccaacctgctgaaggagaaggaaaaactagagttcatcctggcagctcaccgacctgcc
tgcaagatccctgatgacctgggcttcccagaagagatgtctgtggct---tcccttgatctgactgggggcctgccagaggtt---gccacc------
ccg--------------------------------------------------------------------gagtctgaggag---
gccttcaccctgcctctcctcaat---------gaccctgagccc---aagccctcagtggaacctgtcaagagcatc---
agcagcatggagctgaagaccgagccctttgatgacttcctgttcccagcatcatccaggcccagtggctctgagaca---
gcccgctccgtgccagacatggacctatctggg------
tccttctatgcagcagactgggagcctctgcacagtggctccctggggatggggcccatggccacagagctggagcccctgtgcactccggtggtcacctg
tactcccagctgcactgcttacacgtcttccttcgtcttcacctaccccgaggctgactccttccccagctgtgcagctgcccaccgcaagggcagcagca
gcaatgagccttcctctgactcgctcagctcacccacgctgctggccctg
ptroglodytes                             atgatgttctcgggcttcaac------
gcagactacgaggcgtcatcctcccgctgcagcagcgcgtccccggccggggatagcctctcttactaccactcacccgcagactccttctccagcatggg
ttcgcctgtcaacgcgcaggacttctgcacggacctggccgtctccagtgccaacttcattcccacggtcactgccatctcgaccagtccggacctgcagt
ggctggtgcagcccgccctcgtctcctccgtggccccatcgcagaccagagcccctcaccctttcggagtccccacccccctcc---
gctggggcttactccagggctggcgttgtgaagaccatgacaggaggccgagcgcagagcattggcaggaggggcaaggtggaacagttatctccagaaga
agaagagaaaaggagaatccgaagggaaaggaataagatggctgcagccaaatgccgcaaccggaggagggagctgactgatacactccaagcggagacag
accaactagaagatgagaagtctgctttgcagaccgagattgccaacctgctgaaggagaaggaaaaactagagttcatcctggcagctcaccgacctgcc
tgcaagatccctgatgacctgggcttcccagaagagatgtctgtggct---tcccttgatctgactgggggcctgccagaggtt---gccacc------
ccg--------------------------------------------------------------------gagtctgaagag---
gccttcaccctgcctctcctcaat---------gaccctgagccc---aagccctcagtggaacctgtcaagagcatt---
agcagcatggagctgaagaccgagccctttgatgacttcctgttcccagcatcatccaggcccagtggctctgagaca---
gcccgctccgtgccagacatggacctatctggg------
tccttctatgcagcagactgggagcctctgcacagtggctccctggggatggggcccatggccacagagctggagcccctgtgcactccggtggtcacctg
[illegible]
```

- For comparison, the final codon-based alignment PHYLIP files that are used for the analysis are available on OSF (https://osf.io/rxphw/, directory `../Code and data/scripts_files_positive_selection_analysis/step05_aBSREL_input_files/`)

6. <u>Build phylogenetic **species** tree</u>
   - Go to http://timetree.org/ and scroll down to “Build a TimeTree”. In the section “Load a List of Species”, choose a plain text document (.txt) with the full Latin names of the thirteen species (you can use the file provided “thirteenspecies_timetree.txt” available at https://osf.io/rxphw/). Hit “upload”.
   - Inspect the file and download as a Newick file
   - Open the tree file with the text editor or a similar program
   - Remove the branch lengths, i.e., the colon followed by the large number. Do **not** remove the commas, parentheses, or numbers like ‘37’, ‘13’, etc. The examples are highlighted in green below

```
((Carcharodon_carcharias:462.40000000,(Danio_rerio:429.00000000,(Xenopus_tropicalis:351.68654000,
(((Sarcophilus_harrisii:160.00000000,((Bos_taurus:94.00000000,(Mus_musculus:86.38700000,
((Homo_sapiens:5.01052000,Pan_troglodytes:5.36947000)'14':22.42000000,Macaca_mulatta:27.49404000)'13'
:58.38000000)'25':6.80000000)'37':5.18870000,Dasypus_novemcinctus:99.18870000)'36':60.81130000)'35':2
0.06610000,Ornithorhynchus_anatinus:180.06610000)'34':138.88390000,Gallus_gallus:318.95000000)'43':32
.73654000)'33':77.31346000)'51':33.40000000)'50':100.98999000,Petromyzon_marinus:563.38999000);
```

- Shorten the species names e.g., from Homo sapiens to hsapiens, from Danio rerio to drerio, etc.

```
((ccarcharias,(drerio,(xtropicalis,(((sharrisii,((btaurus,(mmusculus,
((hsapiens,ptroglodytes)'14',mmulatta)'13')'25')'37',dnovemcinctus)'36')'35',oanatinus)'34',ggallus)'
43')'33')'51')'50',pmarinus);
```

→ Save as "timetree_bi.nwk"

7. Run exploratory test for positive selection (aBSREL)
   - Open the terminal and cd into the hyphy folder with `cd /path/to/your/hyphyfolder/HYPHY/hyphy`
   - Execute `./HYPHYMP`
   - The program will start running and offering you a selection of tests. Choose **(1) Selection Analyses** (by typing 1 and hitting ENTER)

     NB: The numbering of the tests and where the aBSREL test is to find in HyPHY may depend on/change with the version of HyPhy you are using. Always double-check, also with these instructions, whether the numbering and analyses names you are choosing are correct. **I am using version 2.5.52 here.**
   - Next, choose **(6) [aBSREL] Test for lineage-specific evolution using the branch-site method aBS-REL (Adaptive Branch-Site Random Effects Likelihood).**
   - Supply one of the codon-based alignment PHYLIP files you previously created for one gene by entering the path to the file + filename, e.g., `/path/to/file/TriFusion/out/adcy1_codalign.phy`
   - Upload the tree file you just customized by entering the path + filename, e.g., `/path/to/file/TimeTree/timetree_bi.nwk`
     → hit ENTER and the program will start running
   - Move the output .json file to a folder called "results_logs"; save the progress printed to the terminal while the program runs as a .txt file called gene_aBSREL_log.txt, e.g., adcy1_aBSREL_log.txt

-- Optional – only to recreate the entire analyses as presented in the paper --

8. Clean and organize output data into supplementary data 4
   - Manually create an excel file called "sup_dat_04_pre.xlsx" in a directory called "`../data/raw/`" in the following format:
   - 39 sheets, one sheet for each gene/aBSREL run
   - Each sheet has eight columns called the following: 1st column: Gene, 2nd column: Branch, 3rd column: Omega rate, 4th column: Max. dN/dS, 5th column: Test LRT, 6th column: p-value (uncorrected), 7th column: p-value (Bonferroni), 8th column: p-value (FDR)
   - Enter the gene name/id only in the first row of the first column. It is only necessary for double-checking in R, once you loaded the file into R, that you loaded the correct sheet
   - Enter the branches and nodes in the second column in the following order: pmarinus, ccarcharias, Node3, drerio, Node5, xtropicalis, Node7, ggallus, Node8, oanatinus, Node9, sharrisii, Node11, dnovemcinctus, Node12, btaurus, Node14, mmusculus, Node16, mmulatta, Node17, ptroglodytes, hsapiens
   - Copy-paste the respective information from the log .txt-files and the .json-files into the excel sheet. Leave the column "p-value (FDR)" empty.

- For comparison, a "sup_dat_04_pre.xlsx" file is available on OSF (https://osf.io/rxphw/, directory `../Code and data/scripts_files_positive_selection_analysis/sup_dat_04_pre.xlsx`)
- If not already present, created a directory called "`../data/processed/`" and within that directory, a path called "`files/dnds_fdr_corr/`"
- Run the R script "02hyphy_results_fdr.R". The script results in 38 separate excel files. For comparison those excel files are available on OSF (https://osf.io/rxphw/, directory `../Code and data/data/processed/abagen_dk/files/dnds_fdr_corr/`)
- Combine the 38 single resulting output excel files into one file with 38 sheets, one sheet for one output file, called "sup_dat_04.xlsx". Remove the first "Gene" column from every sheet and add an empty sheet for the gene *MYLK3*. **Move the excel file into the directory "`../data/processed/`".** This will be supplementary data 4 with the final corrected *p*-values which indicate statistically significant positive selection of a branch or node. **You will need this supplementary data for plotting figure 3 (see below).** For comparison, the supplementary data 4 is also available on OSF.

9. Prepare data for plotting of Max. dN/dS values
   - If not already present, create a directory "`../output/figures/`"
   - To plot the Max dN/dS values run the R script "02tree_dnds_visualization.R". It will generate the figure 3 and the supplementary data 5 ("sup_dat_05.xlsx").

-- end

Supplementary references

1. Dowle, M., & Srinivasan, A. (2021). *data.table: Extension of‚data.frame‘*. R package version 1.14.2, https://CRAN.R-project.org/package=data.table, https://github.com/Rdatatable/data.table

2. Firke, S. (2023). *janitor: Simple Tools for Examining and Cleaning Dirty Data.* R package version 2.2.0, https://cran.r-project.org/web/packages/janitor/index.html, https://github.com/sfirke/janitor, https://sfirke.github.io/janitor/

3. Gandrud, C. (2016). *DataCombine: Tools for Easily Combining and Cleaning Data Sets*. R package version 0.2.21, https://CRAN.R-project.org/package=DataCombine, https://github.com/christophergandrud/DataCombine

4. Ooms, J. (2021). *writexl: Export Data Frames to Excel 'xlsx' Format*. R package version 1.4.0, https://CRAN.R-project.org/package=writexl, https://github.com/ropensci/writexl

5. Schauberger, P. & Walker, A. (2022). openxlsx: *Read, Write and Edit xlsx Files.* R package version 4.2.5.2, https://cran.r-project.org/web/packages/openxlsx/index.html, https://github.com/ycphs/openxlsx, https://ycphs.github.io/openxlsx/index.html

6. Slowikowski, K. (2021). *ggrepel: Automatically Position Non-Overlapping Text Labels with 'ggplot2'*. R package version 0.9.1, https://CRAN.R-project.org/package=ggrepel, https://github.com/slowkow/ggrepel/

7. Wickham, H., Hester, J., & Bryan, J. (2022). *readr: Read Rectangular Text Data.* R package version 2.1.2, https://CRAN.R-project.org/package=readr, https://readr.tidyverse.org, https://github.com/tidyverse/readr

8. Wickham, H., & Bryan, J. (2022). *readxl: Read Excel Files*. R package version 1.4.0, https://CRAN.R-project.org/package=readxl, https://readxl.tidyverse.org, https://github.com/tidyverse/readxl

9. Wilke, C. (2020). *cowplot: Streamlined Plot Theme and Plot Annotations for 'ggplot2'*. R package version 1.1.1, https://CRAN.R-project.org/package=cowplot, https://github.com/wilkelab/cowplot

10. Wilke, C. (2021). *ggridges: Ridgeline Plots in 'ggplot2'*. R package version 0.5.3, https://CRAN.R-project.org/package=ggridges, https://github.com/wilkelab/ggridges
