# Supplementary material for: An evolutionary timeline of the oxytocin signaling pathway
Source: Commun Biol. 2024 Apr 17;7:471. doi: 10.1038/s42003-024-06094-9 (PMC11024182; doi:10.1038/s42003-024-06094-9)
Supplement: Supplementary file 3 — Description of Supplementary Materials [file 42003_2024_6094_MOESM3_ESM.docx]

**Description of Additional Supplementary Files**

**File name:** Supplementary data 1 (sheet 1)

**Description:** List of species considered for BLASTp/microsynteny in descending, phylogenetic order

**File name:** Supplementary data 1 (sheet 2)

**Description**: BLASTp thresholds for invertebrates, numeric and boolean

**File name:** Supplementary data 2 (all sheets)

**Description:** BLASTp/microsynteny results for vertebrates for each gene supporting the OT pathway

**File name:** Supplementary data 3

**Description:** List of all genes supporting the OT pathway and i.a. the assigned phylostratum and branch

**File name:** Supplementary data 4

**Description:** Results from the exploratory positive selection analysis

**File name:** Supplementary data 5

**Description:** Raw data underlying figure 3

**File name:** Supplementary data 6

**Description:** Enrichment of ancient genes supporting the OT pathway in different categories and pathways

**File name:** Supplementary data 7

**Description:** Enrichment of medium-aged genes supporting the OT pathway in different categories and pathways

**File name:** Supplementary data 8

**Description:** Enrichment of modern genes supporting the OT pathway in different categories and pathways

**File name:** Supplementary data 9

**Description:** Test statistic results for the cerebral expression analysis

**File name:** Supplementary data 10

**Description:** Differential stability values for the genes supporting the OT pathway

**File name:** Supplementary data 11

**Description:** Raw data underlying figure 4

**File name:** Supplementary data 12

**Description:** Raw data underlying figure 5a

**File name:** Supplementary data 13

**Description:** Raw data underlying figure 5b
