# Supplementary material for: An evolutionary timeline of the oxytocin signaling pathway
Source: Commun Biol. 2024 Apr 17;7:471. doi: 10.1038/s42003-024-06094-9 (PMC11024182; doi:10.1038/s42003-024-06094-9)
Supplement: Supplementary file 5 — Reporting Summary [file 42003_2024_6094_MOESM5_ESM.pdf]

nature portfolio

Corresponding author(s): *Double-blind peer review submissions: write DBPR and your manuscript number here instead of author names.*

Last updated by author(s): *YYYY-MM-DD*

Reporting Summary

Nature Portfolio wishes to improve the reproducibility of the work that we publish. This form provides structure for consistency and transparency in reporting. For further information on Nature Portfolio policies, see our Editorial Policies and the Editorial Policy Checklist.

Statistics

For all statistical analyses, confirm that the following items are present in the figure legend, table legend, main text, or Methods section.

n/a	Confirmed	
☐	☒	The exact sample size (*n*) for each experimental group/condition, given as a discrete number and unit of measurement
☒	☐	A statement on whether measurements were taken from distinct samples or whether the same sample was measured repeatedly
☐	☒	The statistical test(s) used AND whether they are one- or two-sided<br>*Only common tests should be described solely by name; describe more complex techniques in the Methods section.*
☒	☐	A description of all covariates tested
☐	☒	A description of any assumptions or corrections, such as tests of normality and adjustment for multiple comparisons
☐	☒	A full description of the statistical parameters including central tendency (e.g. means) or other basic estimates (e.g. regression coefficient) AND variation (e.g. standard deviation) or associated estimates of uncertainty (e.g. confidence intervals)
☐	☒	For null hypothesis testing, the test statistic (e.g. *F*, *t*, *r*) with confidence intervals, effect sizes, degrees of freedom and *P* value noted<br>*Give P values as exact values whenever suitable.*
☒	☐	For Bayesian analysis, information on the choice of priors and Markov chain Monte Carlo settings
☒	☐	For hierarchical and complex designs, identification of the appropriate level for tests and full reporting of outcomes
☐	☒	Estimates of effect sizes (e.g. Cohen's *d*, Pearson's *r*), indicating how they were calculated

Our web collection on statistics for biologists contains articles on many of the points above.

Software and code

Policy information about availability of computer code

Data collection	No data was collected for this study.
Data analysis	If not stated otherwise, the statistical software R (version 4.2.0) and RStudio (version 2022.7.1.554) were used for analyses and data visualizations (except for fig. 1, fig. 2, parts of fig. 3, and fig. 4a, where Adobe Illustrator 2023 and BioRender were used). The R package “tidyverse” was used to conduct core analyses (see supplementary references for further R packages used). The preprocessing of the AHBA expression data and the methodological report thereof were generated with the abagen toolbox (version 0.1.1). All scripts were custom coded and written (available at https://osf.io/rxphw/). Further (online) software, (online) tools and analysis approaches used for this study include BLASTp, phylostratigraphy, manual microsynteny, NCBI, MUSCLE (version 5.1), PAL2NAL (version 14), TriFusion (version 1.0.1), TimeTree (version 5), aBSREL (version 2.3) from HyPhy (version 2.5.52), FUMA (option GENE2FUNC, including the dataset GTEx v8), and the “ggseg” R package.

For manuscripts utilizing custom algorithms or software that are central to the research but not yet described in published literature, software must be made available to editors and reviewers. We strongly encourage code deposition in a community repository (e.g. GitHub). See the Nature Portfolio guidelines for submitting code & software for further information.

Data

Policy information about availability of data

All manuscripts must include a data availability statement. This statement should provide the following information, where applicable:

- Accession codes, unique identifiers, or web links for publicly available datasets
- A description of any restrictions on data availability
- For clinical datasets or third party data, please ensure that the statement adheres to our policy

Sequences used for the gene age estimations/phylostratigraphy and positive selection analysis were downloaded from https://blast.ncbi.nlm.nih.gov/. The databases for the functional annotation analysis in FUMA are included and available in the FUMA online tool at https://fuma.ctglab.nl/. The AHBA data and cerebral atlas used for this study are included in the Python toolbox abagen. Further information and other download links for the AHBA data are available at https://human.brain-map.org/. All other data used in the analyses not mentioned above is deposited at https://osf.io/rxphw/.

Human research participants

Policy information about studies involving human research participants and Sex and Gender in Research.

Reporting on sex and gender	We used the publicly available data on human brain gene expression from the Allen Institute (Allen Human Brain Atlas "AHBA"). According to the documentation from the Allen Institute, the sample consists of n = 6; 1 female, 5 males; ages 24.0-57.0; three of Caucasian ethnicity, two African American, one Hispanic (https://human.brain-map.org).
Population characteristics	Please refer to the documentation of the original data at https://human.brain-map.org.
Recruitment	Please refer to the documentation of the original data at https://human.brain-map.org.
Ethics oversight	According to the documentation from the Allen Institute, the data collection process complied with the relevant ethical regulations concerning the collection and processing of human post-mortem tissue samples. Consent from the next-of-kin of each donor was obtained. Please also refer to the documentation of the original data at https://human.brain-map.org.

Note that full information on the approval of the study protocol must also be provided in the manuscript.

Field-specific reporting

Please select the one below that is the best fit for your research. If you are not sure, read the appropriate sections before making your selection.

☐ Life sciences ☐ Behavioural & social sciences ☒ Ecological, evolutionary & environmental sciences

For a reference copy of the document with all sections, see nature.com/documents/nr-reporting-summary-flat.pdf

Ecological, evolutionary & environmental sciences study design

All studies must disclose on these points even when the disclosure is negative.

Study description	Oxytocin is a neuropeptide associated with both psychological and somatic processes like parturition and social bonding. Although oxytocin homologs have been identified in many species, the evolutionary timeline of the entire oxytocin signaling gene pathway has yet to be described. Using protein sequence similarity searches, microsynteny, and phylostratigraphy, we assigned the genes supporting the oxytocin pathway to different phylostrata based on when we found they likely arose in evolution. We show that the majority (64%) of genes in the pathway are 'modern'. Most of the modern genes evolved around the emergence of vertebrates or jawed vertebrates (540 - 530 million years ago, 'mya'), including OXTR, OXT and CD38. Of those, 45% were under positive selection at some point during vertebrate evolution. We also found that 18% of the genes in the oxytocin pathway are 'ancient', meaning their emergence dates back to cellular organisms and opisthokonta (3500 - 1100 mya). The remaining genes (18%) that evolved after ancient and before modern genes were classified as 'medium-aged'. Functional analyses revealed that, in humans, medium-aged oxytocin pathway genes are highly expressed in contractile organs, while modern genes in the oxytocin pathway are primarily expressed in the brain and muscle tissue.
Research sample	Proteomes and genomes from 26 invertebrate species (e.g., E. coli, S. cerevisiae, P. caudatus, A. rubens, C. intestinalis) and 13 vertebrate species (e.g., P. marinus, X. tropicalis, M. musculus, H. sapiens) representing major evolutionary branches and lineages in order to cover the entire evolutionary timeline, all available at https://www.ncbi.nlm.nih.gov/. Public data set "Allen Human Brain Atlas Data" (AHBA) on cerebral gene expression including n = 6 post-mortem human brain specimens; 1 female, 5 males; ages 24.0-57.0; three of Caucasian ethnicity, two African American, one Hispanic; https://human.brain-map.org. AHBA was chosen because it is a unique resource for high-resolution, fine-parcellated human cerebral gene expression data. The FUMA online tool was used which includes human expression data from the GTEx sample. Information on FUMA can be found in Watanabe et al., 2017 (https://doi.org/10.1038/s41467-017-01261-5), and information regarding GTEx v8 RNA-seq data can be obtained from The GTEx Consortium et al., 2015 (https://doi.org/10.1126/science.1262110).
Sampling strategy	26 invertebrate and 13 vertebrate species were chosen because they cover the major evolutionary branches from cellular organisms

Sampling strategy	to the modern human, with at least two species per branch. The sample size in the AHBA data set was predetermined, as well as the sampling characteristics for the GTEx data.
Data collection	No data for this study was collected, only public data repositories were used.
Timing and spatial scale	No data for this study was collected, only public data repositories were used.
Data exclusions	Not applicable (see above).
Reproducibility	In order to ensure reproducibility, all code and data is public and available at https://osf.io/rxphw/.
Randomization	Not applicable (see above).
Blinding	Not applicable (see above).

Did the study involve field work? ☐ Yes ☒ No

Reporting for specific materials, systems and methods

We require information from authors about some types of materials, experimental systems and methods used in many studies. Here, indicate whether each material, system or method listed is relevant to your study. If you are not sure if a list item applies to your research, read the appropriate section before selecting a response.

Materials & experimental systems

n/a	Involved in the study
☒	☐ Antibodies
☒	☐ Eukaryotic cell lines
☒	☐ Palaeontology and archaeology
☒	☐ Animals and other organisms
☒	☐ Clinical data
☒	☐ Dual use research of concern

Methods

n/a	Involved in the study
☒	☐ ChIP-seq
☒	☐ Flow cytometry
☒	☐ MRI-based neuroimaging
